# Cannabigerol modulates α_2_-adrenoceptor and 5-HT_1A_ receptor-mediated electrophysiological effects on dorsal raphe nucleus and locus coeruleus neurons and anxiety behavior in rat

**DOI:** 10.3389/fphar.2023.1183019

**Published:** 2023-05-25

**Authors:** Aitziber Mendiguren, Erik Aostri, Irati Rodilla, Iker Pujana, Ekaterina Noskova, Joseba Pineda

**Affiliations:** Department of Pharmacology, Faculty of Medicine and Nursing, University of the Basque Country (UPV/EHU), Leioa, Spain

**Keywords:** dorsal raphe nucleus, slice, firing, noradrenaline, locus coeruleus, cannabigerol, anxiety, serotonin

## Abstract

The pharmacological profile of cannabigerol (CBG), which acid form constitutes the main precursor of the most abundant cannabinoids, has been scarcely studied. It has been reported to target α_2-_adrenoceptor and 5-HT_1A_ receptor. The locus coeruleus (LC) and the dorsal raphe nucleus (DRN) are the main serotonergic (5-HT) and noradrenergic (NA) areas in the rat brain, respectively. We aimed to study the effect of CBG on the firing rate of LC NA cells and DRN 5-HT cells and on α_2_-adrenergic and 5-HT_1A_ autoreceptors by electrophysiological techniques in male Sprague-Dawley rat brain slices. The effect of CBG on the novelty-suppressed feeding test (NSFT) and the elevated plus maze test (EPMT) and the involvement of the 5-HT_1A_ receptor was also studied. CBG (30 μM, 10 min) slightly changed the firing rate of NA cells but failed to alter the inhibitory effect of NA (1–100 µM). However, in the presence of CBG the inhibitory effect of the selective α_2_-adrenoceptor agonist UK14304 (10 nM) was decreased. Perfusion with CBG (30 μM, 10 min) did not change the firing rate of DRN 5-HT cells or the inhibitory effect of 5-HT (100 μM, 1 min) but it reduced the inhibitory effect of ipsapirone (100 nM). CBG failed to reverse ipsapirone-induced inhibition whereas perfusion with the 5-HT_1A_ receptor antagonist WAY100635 (30 nM) completely restored the firing rate of DRN 5-HT cells. In the EPMT, CBG (10 mg/kg, i.p.) significantly increased the percentage of time the rats spent on the open arms and the number of head-dipping but it reduced the anxiety index. In the NSFT, CBG decreased the time latency to eat in the novel environment but it did not alter home-cage consumption. The effect of CBG on the reduction of latency to feed was prevented by pretreatment with WAY100635 (1 mg/kg, i.p.). In conclusion, CBG hinders the inhibitory effect produced by selective α_2_-adrenoceptor and 5-HT_1A_ receptor agonists on the firing rate of NA-LC and 5-HT-DRN neurons by a yet unknown indirect mechanism in rat brain slices and produces anxiolytic-like effects through 5-HT_1A_ receptor.

## 1 Introduction

*Cannabis sativa* plant contains more than 120 phytocannabinoids including psychoactive and non-psychoactive compounds (Turner et al., 2017). Several studies have been performed to characterize the pharmacological effects of the non-psychoactive cannabinoids. The best characterized one is cannabidiol (CBD), which has been shown to target the G_i/o_ protein-coupled 5-HT_1A_ receptor to mediate its principal therapeutical effects, such as anxiolytic or antiepileptic effects (De Gregorio et al., 2019; Silvestro et al., 2020). Another non-psychoactive phytocannabinoid is cannabigerol (CBG), which acid form has been identified as the main precursor of the most abundant cannabinoids. CBG has been suggested to constitute a potential drug for disease treatment since it stimulates appetite (Brierley et al., 2016) and shows analgesic, anti-inflammatory (di Giacomo et al., 2020), antiemetic (Rock et al., 2011) or anxiolytic (Zagzoog et al., 2020) effects in rodents. However, to date little is known about its pharmacological profile, although it has been postulated to be between that of Δ^9^-THC and CBD (Nachnani et al., 2021).

The main nucleus in the central nervous system (CNS) enriched with noradrenergic (NA) cells is the locus coeruleus (LC), which activity is regulated, among others, by the G_i/o_ protein-coupled α_2-_adrenoceptor (Aghajanian and Wang, 1987). The dorsal raphe nucleus (DRN) is the principal 5-HT nucleus in the rat brain. 5-HT cells of the DRN expresses the G_i/o_ protein-coupled 5-HT_1A_ autoreceptor and its activation results in the inhibition of the firing activity of 5-HT cells via G protein-coupled inwardly rectifying potassium channels (GIRKs) (Courtney and Ford, 2016). Both monoaminergic nuclei play a pivotal role in the regulation of physiological processes and pathological conditions including sleep-wake phase, arousal, pain, anxiety and depression (Aston-Jones et al., 1999; Michelsen et al., 2007; Lowry et al., 2008; Monti, 2010; Takahashi et al., 2010; Felippotti et al., 2011; Lin et al., 2011; Campion et al., 2016; Morris et al., 2020; Suárez-Pereira et al., 2022).

Several studies have demonstrated that psychoactive and non-psychoactive cannabinoids regulate monoaminergic systems (Mendiguren et al., 2018). On the one hand, CB_1_ receptor agonists and/or antagonists modulate the neuronal activity of DRN 5-HT cells or LC NA cells *in vivo* and *in vitro* (Gobbi et al., 2005; Muntoni et al., 2006; Mendiguren and Pineda, 2009; Bambico et al., 2012; Mendiguren et al., 2018). On the other hand, CBD reduces the firing rate of DRN 5-HT cells *in vivo* (De Gregorio et al., 2019) and modulates 5-HT_1A_ receptor-mediated effects on the firing rate of DRN 5-HT cells *in vitro* (Mendiguren et al., 2022). Furthermore, several *in vivo* studies have reported that the 5-HT or NA systems are involved in the anxiolytic (De Gregorio et al., 2019; Warren et al., 2022) and antidepressant effects of cannabinoids (Gobbi et al., 2005; Zanelati et al., 2010; Kirilly et al., 2013; Linge et al., 2016; Sartim et al., 2016; Sales et al., 2018). However, the *in vivo* and *in vitro* effects of the non-psychoactive cannabinoid CBG have been scarcely studied. *In vitro* data have shown that CBG targets α_2-_adrenoceptor and 5-HT_1A_ receptor (Cascio et al., 2010; Rock et al., 2011). Thus, a single study performed by binding techniques in mouse brain membranes revealed that CBG behaves as potent α_2-_adrenoceptor agonist and moderately potent 5-HT_1A_ receptor antagonist (Cascio et al., 2010). In addition, few data exist on the pharmacological effects (i.e., anxiolytic effect) of CBG in rodent. Moreover, the involvement of 5-HT_1A_ receptor in the *in vivo* effects of CBG have not been investigated yet, even though it has been reported that CBG exerts 5-HT_1A_ receptor-mediated neuroprotective effects *in vitro* (Echeverry et al., 2021).

Therefore, considering that the α_2_-adrenoceptor is abundant in the LC and that the 5-HT_1A_ receptor is widely distributed in the DRN, the aim of our work was to characterize the effect of CBG on the firing rate of NA and 5-HT cells and on the 5-HT_1A_ and α_2-_ autoreceptors activation by electrophysiological techniques in rat brain slices from the LC and DRN. Furthermore, we studied the effect of CBG on anxiety-like behavior by the elevated plus maze test (EPMT) and the novelty-suppressed feeding test (NSFT) and the putative involvement of the 5-HT_1A_ receptor.

## 2 Materials and methods

### 2.1 Animals

Male Sprague-Dawley (200–300 g) rats (total, n = 176; behavioral tests, n = 118; and electrophysiological assays, n = 58) were kept under controlled environmental conditions (22 °C, 12L:12D schedule, 65–70% humidity, food and water *ad libitum*). The experiments were conducted following the European Directive on the protection of animals for scientific purposes (2010/63/EU). All the procedures were accepted by the Institutional Ethical Committee for Research and Teaching of the University of the Basque Country (UPV/EHU, Spain) and the Department of Sustainability and Natural Environment of Provincial Council from Bizkaia (ref. CEEA M20-2018-025 and CEEA M20-2018-262). A minimum number of animals was used and we made an effort to avoid animal suffering.

### 2.2 Electrophysiological experiments

#### 2.2.1 Brain slicing

The rat was anaesthetized with chloral hydrate (400 mg/kg i.p.) and then brain was extracted after decapitation. The tissue was transferred to an ice-cold artificial cerebrospinal fluid (ACSF), in which NaCl was replaced by sucrose to enhance neuronal survival. Coronal slices containing the LC or the DRN of 500–600 µm thickness were cut by a vibratome and they were allowed to recover from the slicing for 2 h. Then, the brainstem sections were placed in a custom-made modified Haas-type interface chamber. The slice was continuously perfused with ACSF (flow rate: 1.5 mL/min, 33 °C), which was composed of NaCl 130 mM, KCl 3 mM, NaH_2_PO_4_ 1.25 mM, MgSO_4_ 2 mM, CaCl_2_ 2 mM, NaHCO_3_ 20 mM and D-glucose 10 mM bubbled with 95% O_2_/5% CO_2_ (pH = 7.34) (Mendiguren and Pineda, 2009).

#### 2.2.2 Extracellular recordings

Single-unit extracellular recordings of LC NA neurons and DRN 5-HT neurons were made as previously described (Mendiguren and Pineda, 2009; Medrano et al., 2017; Mendiguren et al., 2022). An Omegadot glass micropipette was prepared with a horizontal pipette puller and filled with NaCl (0.05 M). Then, the tip was broken to a diameter of 2–5 µm for a final resistance of 3–5 MΩ. The electrode was placed under binocular microscope in the recording area (LC or DRN). The LC nucleus was visually identified in the rostral pons as a dark oval area on the lateral borders of the central gray and the fourth ventricle, anterior to the genu of the facial nerve. The DRN was localized visually as a dark area in the ventromedial part of the periaqueductal gray. The extracellular signal was filtered and amplied through a high-input impedance amplifier. Then, an oscilloscope and audio analyzer were used to monitor the signal. Individual (single-unit) neuronal spikes were discriminated from the background noise with a window discriminator. The firing rate was analyzed by a PC-based custom-made software, which generated consecutive histogram bars representing the accumulated number of spikes in 10 s. NA LC cells were identified by their spontaneous and regular discharge activity, slow firing rate, and long-lasting biphasic positive-negative waveforms (Andrade and Aghajanian, 1984; Medrano et al., 2017). The effect of GABA (1 mM) was used as a control for the perfusion system and to normalize the inhibitory effects of NA. We only selected the cells that were initially inhibited by GABA (1 mM, 1 min). One animal was used for each experiment and only one cell from each slice was recorded. DRN 5-HT cells were selected based on electrophysiological and pharmacological criteria. The electrophysiological features to identify 5-HT cells were the following: a regular discharging pattern, a slow firing rate and a long-lasting biphasic positive-negative waveform (2 ms). As a pharmacological criteria, the response to short perfusion of 5-HT (50–100 μM, 1 min) was used (Aghajanian and Lakoski, 1984; Hajós et al., 1996; Mendiguren and Pineda, 2009; Mendiguren et al., 2022). Only the neurons that showed the mentioned electrophysiological features and were inhibited by 5-HT were selected for the study. In all cases, the firing rate was driven by perfusion with the α_1_-adrenoceptor agonist phenylephrine (PE, 15 μM) because in slices from the DRN the NA excitatory afferents are cut and 5-HT cells fail to discharge spontaneously (Mendiguren and Pineda, 2009; Mendiguren et al., 2022).

#### 2.2.3 Experimental design

To study the effect of CBG (30 µM) on the firing rate of LC NA cells and on the PE-driven firing activity of DRN 5-HT cells, we first perfused the vehicle of the drug (DMSO ≤0.1%) and then the cannabinoid for 10 min. The effect of CBG on α_2_-adrenoceptor mediated inhibition of the firing rate was studied by perfusing increasing concentrations of NA (1–100 μM, x3, 1 min each) or the more selective α_2_-adrenoceptor agonist UK14304 (1 nM and 10 nM) in the absence and in the continuous presence of CBG. UK14304 was applied until a maximal inhibition plateau was reached within 10 min of drug perfusion. Unlike NA, which is easily washed out from the slice, experiments of UK14304 in the absence or the presence of CBG were performed in NA cells from different slices. To avoid the influence that changes of the firing rate produced by CBG may have on the quantification of the effects of NA on the firing rate, GABA (1 mM, 1 min) was administrated before performing the concentration-effect curves in the absence and the presence of CBG to normalize the inhibitory effects of NA.

The effect of CBG (30 µM) on the inhibition of the firing rate elicited by 5-HT_1A_ receptor agonists was investigated by studying and comparing the inhibitory responses to application of the endogenous ligand 5-HT (100 μM, 1 min) or the more selective 5-HT_1A_ receptor agonist ipsapirone (100 nM, 10 min) in the absence or the presence of the cannabinoid in DRN 5-HT cells. We also studied whether CBG (30 μM) could restore the firing rate of previously inhibited 5-HT cells and mimick the effect of a competitive 5-HT_1A_ receptor antagonist by perfusing the cannabinoid for 10 min. Finally, after administration of CBG the selective 5-HT_1A_ receptor antagonist WAY100635 (30 nM) was perfused in the same 5-HT cell to restore the firing activity.

### 2.3 Behavioral assays

#### 2.3.1 Elevated plus maze test

The EPM consisted of a cross-shaped, elevated platform with two open (50 cm long x 10 cm wide) and two closed arms (50 cm long x 10 cm wide). Each rat was placed in the central platform facing to the open arm and its behavior was recorded for 5 min. Arm entries were considered as introduction of four paws into the arm. The number of entries and the time spent in the open arms were measured. The frequency of the following ethological parameters was also observed: head-dipping (animal sticking the head toward the floor from the open arm) and rearing (vertical standing of rodent on two hind legs) (Walf and Frye, 2007). Increases of number of entries, time spent in the open arms and the number of head-dipping have been shown to indicate an anxiolytic-like effect (Griebel et al., 1997) while changes in rearing have been suggested to reflect alteration of motor activity (Cruz et al., 1994).

#### 2.3.2 Novelty-suppressed feeding test

NSFT assesses the ability of the animal to resolve a conflict between a novel context that induces heightened anxiety and a drive to approach an appetitive stimulus. Thereby it can be used for evaluating the potential anxiolytic effect of drugs. Novel environment consisted of a clean 100 cm × 100 cm × 40 cm box with open anxiogenic arena. The ground was covered with wooden bedding, which was changed after each single animal experiment. A small piece of rat chow was positioned in the center of the arena onto a circular filter paper, which was illuminated (1000 lm, peripheral intensity 800 lm). The surrounding environment remained lightless. The rat underwent a food restriction period of 24 h with free access to water. After habituation to the room (at least 90 min), the animal was introduced in a corner of the open field and the time it took to eat from the highly-illuminated food was recorded (latency to eat). Longer latency to eat indicates higher level of anxiety-like-behavior. Anxiolytic drugs decrease the latency to feed in food-deprived rats exposed to a novel environment (Shephard and Broadhurst, 1982). An experimental cut off time of 10 min was set. Immediately after, the animal was returned back to its home-cage, where it was provided with a weighed amount of food. After 10 min the amount of food ingested by each rat was determined by weighing the remaining rat chow (home-cage food consumption). Both latency to eat in the home-cage and home-cage food consumption were measured to assure that the tested drugs did not alter food intake since cannabinoids could have hyperphagic effects (Williams and Kirkham, 2002; Nachnani et al., 2021).

#### 2.3.3 Experimental design

CBG (3–10 mg/kg, i.p.) was administrated 60 min prior to carry out the test, according to previous behavioral and pharmacokinetics studies (Deiana et al., 2012; Zagzoog et al., 2020) (CBG group). The control group was injected with the corresponding volume of the vehicle of CBG (3 or 10 mL/kg of 5% cremophor, 5% ethanol, 90% saline) 60 min prior to the test. As no differences were observed in the tested effects among the groups injected with different volumes of the vehicle, all the data from the vehicle-injected animals were gathered in the same group for further comparison analysis. Finally, to study the receptor involved in these effects, some animals received an injection of the 5-HT_1A_ receptor antagonist WAY100635 (1 mg/kg, i.p.) 30 min before administration of CBG (10 mg/kg, i.p.) (WAY100635 + CBG group) or the vehicle of CBG (WAY100635 group).

### 2.4 Drugs

γ-Aminobutyric acid (GABA), 5-Bromo-6-(2-imidazolin-2-ylamino)quinoxaline tartrate (UK14304), (−) Cannabigerol (CBG), 2-[4-[4-(2-pyrimidinyl)-1-piperazinyl]butyl]-1,2-benzisothiazol-3(2H)-one-1,1-dioxide (ipsapirone) and phenylephrine (PE) hydrochloride were purchased from Tocris (Bristol, United Kingdom). 5-hydroxytryptamine (5-HT), noradrenaline (NA) and N-[2-[4-(2-Methoxyphenyl)-1-piperazinyl]ethyl]-N-2-pyridinylcyclohexanecarboxamide (WAY100635) were purchased from Sigma (St Louis, MO, United States).

For electrophysiological assays, stock solutions of ipsapirone, UK14304 and CBG were prepared in dimethylsulphoxide (DMSO), and those of GABA, 5-HT, NA, PE, and WAY100635 in milliQ water. Final solutions were freshly prepared and diluted in ACSF for the desired concentration. Equivalent maximal concentrations of the vehicles in which the drugs were dissolved were applied as a control. The maximal concentration of DMSO in the ACSF was ≤0.1%.

For behavioral studies, CBG was dissolved in a mixture of 5% cremophor, 5% ethanol and 90% saline (0.9% NaCl). WAY100635 was dissolved in 0.9% NaCl (saline).

### 2.5 Data analysis

In electrophysiological experiments, the effect of CBG on the firing rate of NA LC cells and on 5-HT DRN cells was measured at the time of the maximal change in the firing rate after administration of the cannabinoid, which was quantified as the percentage change from the basal firing rate. The inhibitory effects of increasing concentrations of NA on the firing rate of LC cells were normalized to GABA (1 mM, 1 min)-induced inhibition. To construct concentration-effect curves for NA, fitting analysis was performed to obtain the best simple non-linear fit to the following three-parameter logistic equation: E = E_max_/1 + (EC_50_/A)^n^ x 100, where [A] is the concentration of NA, E is the effect on the firing rate induced by NA, E_max_ is the maximal inhibitory effect, EC_50_ is the concentration of the agonist required to promote the 50% of the E_max_ and *n* represents the slope factor of the curve. From this analysis EC_50_, E_max_ and *n* values were calculated. For comparison purposes, EC_50_ values were converted and expressed as the negative logarithm values (pEC_50_, M), which adjusted the variable to a Gaussian distribution.

The effect of 5-HT was calculated by integrating the firing rate values (spikes/10 s, 60 s) after 5-HT application in the absence and in the presence of CBG while the effect of the selective 5-HT_1A_ receptor or α_2_-adrenoceptor agonists was measured by calculating the maximal change in the firing rate within 10 min of drug perfusion. These values were subtracted to the firing rate value (spikes/10 s) before application of the 5-HT_1A_ receptor agonists and then quantified as the percentage change from the basal firing rate.

In behavioral studies, the percentage of time the rats spent on the open arms ([seconds on the open arms]/[300 s] × 100), the percentage of open arm entries ([open entries]/[total entries] × 100) and the anxiety index were calculated in the EPMT for comparisons between CBG and vehicle-treated groups. Anxiety index was expressed as follows: AI = 1 − ([time spent on the open arms/test duration] + [entries into the open arms/total number of entries]/2). Values range from 0 to 1, where an increase in the index expresses higher anxiety-like behavior (Cohen et al., 2013). To study the effect of CBG on the NSFT, the time latency to feed in the novel environment (s), home-cage consumption (g) and latency to eat in the housing cage (s) were measured both in the CBG-treated group and in the matched vehicle-treated group. To characterize the influence of WAY100635 administration on CBG-induced effects, changes in the latency to feed, home-cage consumption and latency to eat in the housing cage produced by the cannabinoid were studied both in the absence (vehicle) and the presence of the 5-HT_1A_ receptor antagonist. The effects of CBG in the absence of WAY100635 were calculated as the percentages of latency to eat, home-cage consumption and latency to feed in the housing cage after CBG administration with respect to the mean value in the control group (vehicle-treated group). The effects of CBG in the presence of WAY100635 were estimated as the percentages of the latency to eat, home-cage consumption and latency to feed in the housing cage after CBG and WAY100635 administration with respect to the mean value in its control group (WAY100635-treated group).

Data are given as mean ± standard error of the mean (SEM). Analysis of the results were done by Graph Pad Prism. For statistical analysis, paired Student’s *t*-test was performed when the effects before and after drug application were compared within the same cell, and by a two-sample Student’s *t*-test when two independent experimental conditions were compared. One-way analysis of variance (ANOVA) followed by the Bonferroni’s multiple comparison poshoc test was used to compare more than two independent groups. The non-parametric Kruskal–Wallis test was used when the criteria for the parametric statistics were not met (frequency of head-dipping and rearing). Subsequently, appropriate paired comparisons were performed using Mann-Whitney *U* test*.* The level of significance was set at *p* < 0.05.

## 3 Results

### 3.1 Effect of CBG on the firing rate of NA cells and on α_2_-adrenoceptor agonist-induced inhibition of the firing activity of LC NA cells

**FIGURE 1:**
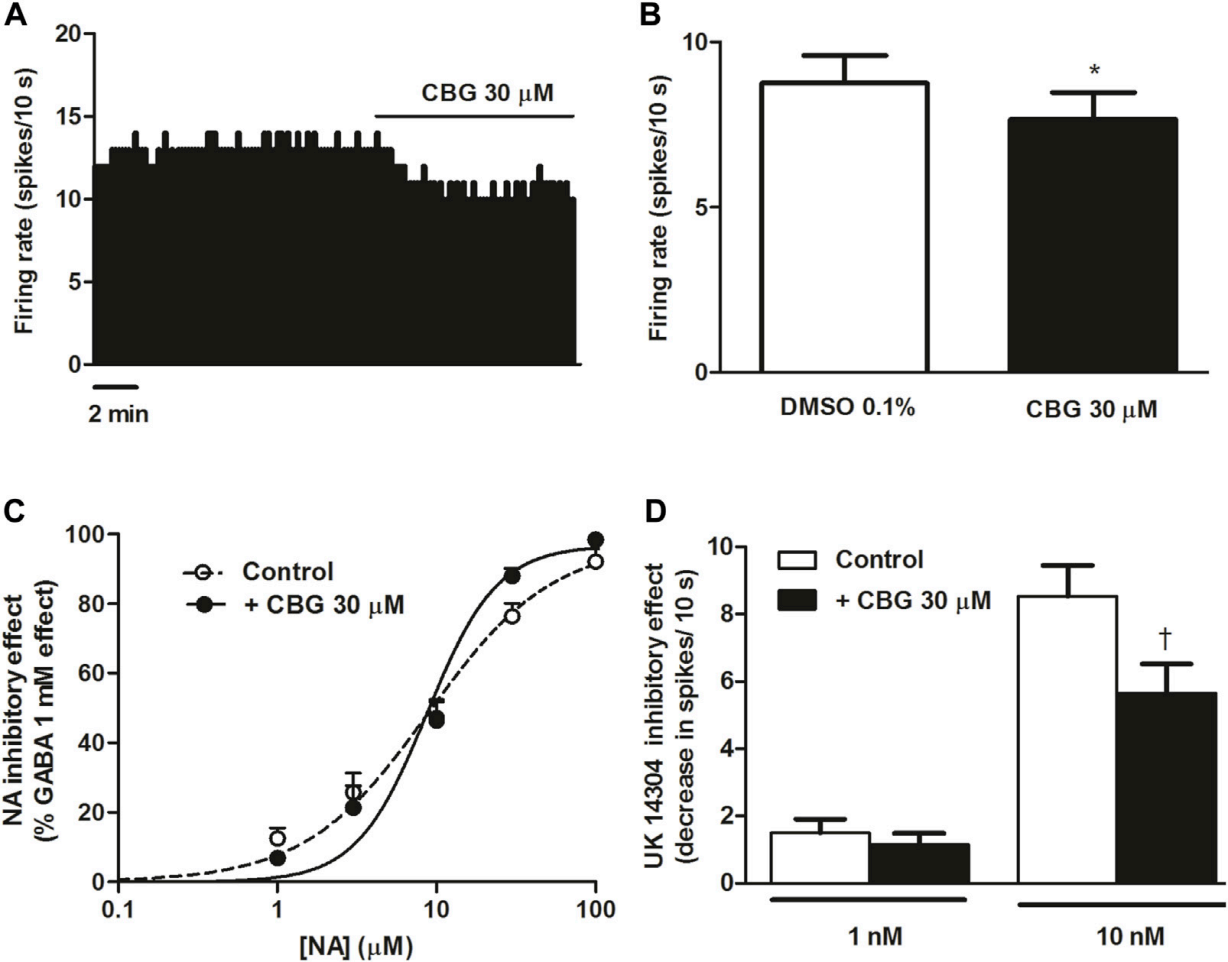
Effect of CBG on the firing rate of LC NA cells and on α_2_-adrenoceptor agonist-induced inhibition **(A)** Representative example of the firing rate recording of LC neuron, which represents the effect of CBG (30 μM) on the basal firing rate. The vertical line indicates the number of spikes recorded every 10 s and the horizontal line shows the period of drug application. **(B)** Bar histograms showing the firing rate (mean ± SEM) in the absence and the presence of CBG (30 μM) (*n* = 16) **(C)** Concentration-effect curves for NA in the absence (non-filled circles, *n* = 6) and the presence of CBG (30 μM) (filled circles, *n* = 6). The horizontal axis shows the concentration of NA in a semi-logarithmic scale. The vertical axis indicates the reduction in the firing rate of LC neurons as the percentage of the inhibitory effect of GABA (1 mM). Data points are the mean ± SEM at each NA concentration obtained from *n* cells. The line through the data is the theoretical curve constructed from the mean of the individual concentration-effect curve parameters, as estimated by nonlinear regression. The parameters of the concentration-effect curve for NA in the absence (control) and the presence of CBG (30 μM) were the following: Emax (97.4 ± 1.9 vs*.* 96.8 ± 2.2%), pEC_50_ (5.05 ± 0.07 M vs*.* 5.06 ± 0.07 M) and EC_50_ (8.93 µM vs*.* 8.70 µM) (*n* = 6). **(D)** Bar histograms showing the inhibitory effect (decrease in spikes/10 s; mean ± SEM) of UK14304 in the absence (control) (1 nM, *n* = 6 and 10 nM, *n* = 6) and the presence of CBG (30 μM) (1 nM, *n* = 9 and 10 nM, *n* = 8). *p < 0.005 compared to the firing rate before CBG (30 μM) administration by a paired Student’s *t*-test. †*p* < 0.05 compared to the inhibitory effect induced by UK14304 (10 nM) in the absence of CBG (30 μM) by an unpaired Student’s *t*-test.

CBG has been shown to be a potent agonist at the α_2_-adrenoceptor in binding assays performed in brain mouse membranes (Cascio et al., 2010). Therefore, we first studied the effect of CBG on the firing rate of NA cells in the LC. Administration of CBG (30 μM, 10 min) slightly decreased the firing rate of LC cells (FR before CBG = 0.88 ± 0.08 Hz vs*.* FR after CBG = 0.77 ± 0.08 Hz, *n* = 16, *p* < 0.005) (Figures 1A, B). The inhibitory effect of CBG was 12.8 ± 3.8%, suggesting that CBG did not behave as a full agonist at the α_2_-adrenoceptor in LC neurons. Perfusion with the vehicle of CBG (DMSO 0.1%, 10 min), which was administrated before the cannabinoid, failed to alter the firing rate of NA cells (FR before: 0.85 ± 0.09 Hz vs*.* FR after: 0.88 ± 0.08 Hz, *n* = 16) (Figure 1B). In order to study whether CBG (30 µM) changed the effect of α_2_-adrenoceptor agonists on the neuronal activity of LC neurons, we tested the effect of the cannabinoid on NA and UK14304-induced inhibition of the firing rate of NA cells. Increasing concentrations of NA (1–100 μM, x3, 1 min each) inhibited the neuronal activity of LC cells in a concentration-dependent manner with an EC_50_ value of 8.93 µM (*n* = 6), which was consistent with that previously reported in LC brain slices (Grandoso et al., 2005). Complete inhibition of the firing rate of NA cells was achieved at the highest concentration of NA (E_max_ = 97.4 ± 1.9%, *n* = 6) (Figure 1C). Continuous perfusion with CBG (30 µM) failed to change the inhibitory effect of NA (1–100 μM, 1 min each). Thus, in the presence of CBG, the EC_50_ value of the concentration-effect curve for NA was 8.70 µM and the E_max_ = 96.8 ± 2.2% (*n* = 6) (Figure 1C). However, administration of CBG (30 µM) reduced the maximal inhibitory effect of UK14304 (10 nM, 5–10 min) (E_max_ in the absence of CBG = 100%, *n* = 6 vs*.* E_max_ in the presence of CBG = 85.9 ± 7.6%, *n* = 8). Thus, the decrease of the firing rate induced by UK14304 (10 nM) in the absence of CBG was significantly higher than that in the presence of CBG (decrease of the FR in the absence of CBG: 0.85 ± 0.09 Hz, *n* = 6 vs*.* decrease of the FR in the presence of CBG: 0.56 ± 0.09 Hz, *n* = 8, *p* < 0.05) (Figure 1D). However, CBG did not change the inhibitory effect of a lower concentration of UK14304 (1 nM, 5–10 min) (0.15 ± 0.04 Hz, *n* = 6 vs*.* 0.11 ± 0.03 Hz, *n* = 9) (Figure 1D).

### 3.2 Effect of CBG on the firing rate of 5-HT cells and on 5-HT_1A_ receptor agonist-induced inhibition of the firing activity of DRN 5-HT cells

**FIGURE 2:**
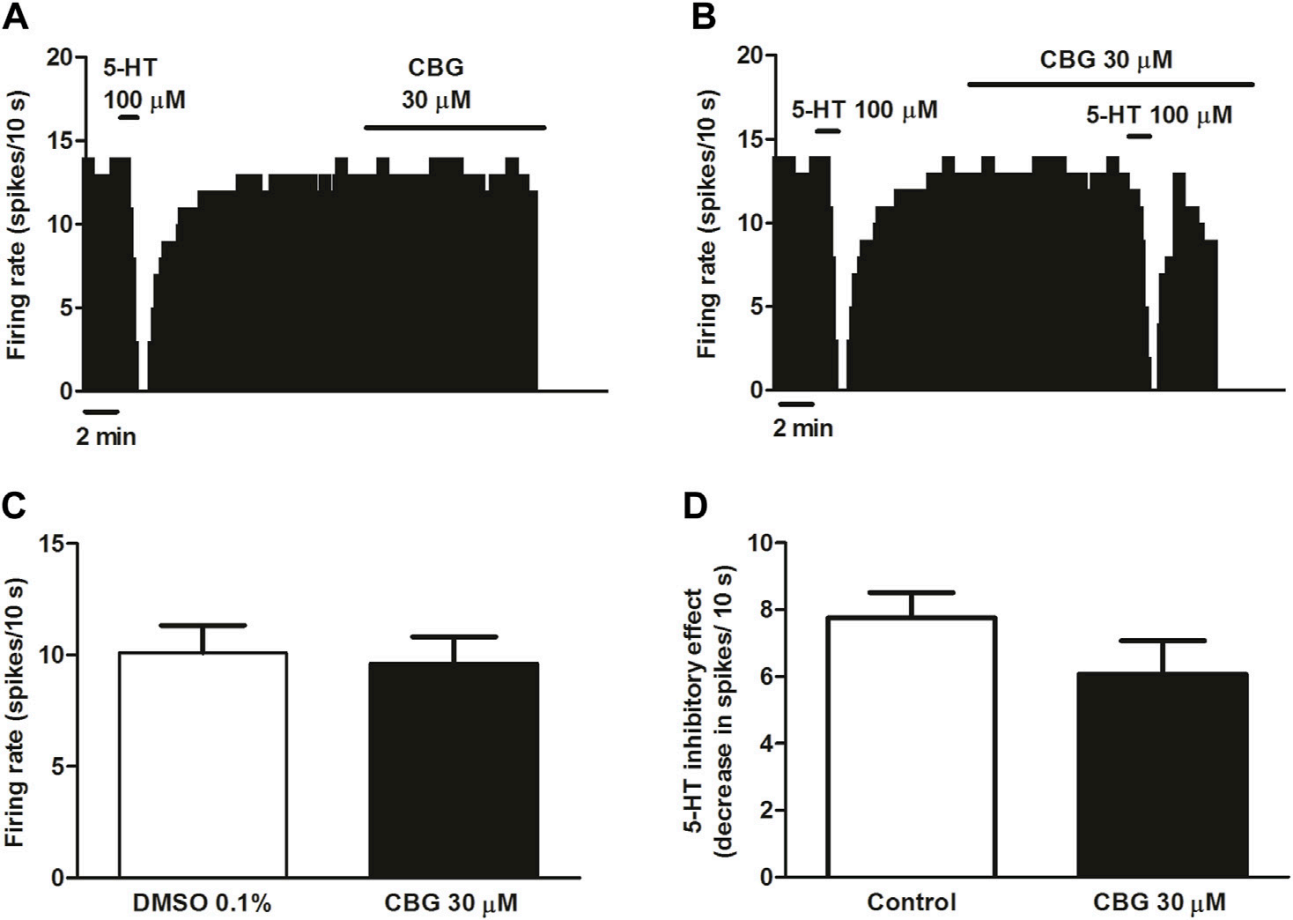
Effect of CBG on the firing rate of 5-HT cells and on 5-HT-induced inhibition of the firing activity of DRN 5-HT cells **(A,B)** Representative examples of firing rate recordings from DRN cells, which show the effect of CBG (30 μM) on the firing activity of 5-HT neurons **(A)** and the effect of CBG (30 μM) on 5-HT (100 μM)-induced inhibition **(B)**. The vertical lines refer to the integrated firing rate values (spikes per 10 s) and the horizontal lines represent the time scale. Drugs were perfused at the concentration and for the time indicated by the horizontal bars. **(C)** Bar histograms showing the firing rate of 5-HT cells (mean ± SEM) before (*n* = 9) and after perfusion with CBG (30 μM) (*n* = 9). **(D)** Bar histograms showing the inhibitory effect (decrease in spikes/10 s; mean ± SEM) induced by 5-HT (100 µM) in the absence (*n* = 9) and the presence of CBG (30 μM) (*n* = 9).

The non-psychoactive cannabinoid CBG has been shown to be a moderate 5-HT_1A_ receptor antagonist in binding assays by blocking 8-OH-DPAT effects in brain mouse membranes (Cascio et al., 2010). In a recent work, we have demonstrated that the best-studied non-psychoactive cannabinoid CBD hinders the effects of 5-HT_1A_ receptor agonists without altering the firing rate of DRN 5-HT cells in rat brain slices (Mendiguren et al., 2022). Therefore, in order to study the effect of CBG on the neuronal activity of 5-HT cells and on the 5-HT_1A_ receptor in the DRN we followed the same pharmacological procedure. Perfusion with CBG (30 μM, 10 min) failed to change the basal firing rate of DRN 5-HT cells (FR before CBG: 1.01 ± 0.13 Hz vs*.* FR after CBG: 0.96 ± 0.12 Hz, *n* = 9) (Figures 2A, C). To test whether the cannabinoid regulated 5-HT_1A_ receptor-mediated effects in the DRN we administrated 5-HT (100 µM) in the absence and in the continuous presence of CBG (30 µM). As expected, administration of 5-HT (100 μM, 1 min) significantly inhibited the firing rate of DRN 5-HT cells (Figures 2B, D). Perfusion with CBG (30 μM, 10 min) did not change the inhibitory effect of 5-HT (100 μM, 1 min) (Figures 2B, D). Thus, in the presence of CBG (30 μM, 10 min) administration of 5-HT (100 μM, 1 min) significantly inhibited the firing rate of DRN 5-HT cells (FR before 5-HT: 0.96 ± 0.12 Hz vs*.* FR after 5-HT: 0.36 ± 0.17 Hz, *n* = 9, *p* < 0.005) and the inhibition was not different from that induced by 5-HT in the absence of CBG (FR before 5-HT: 0.99 ± 0.1 Hz vs*.* FR after 5-HT: 0.21 ± 0.05 Hz, *n* = 9, *p* < 0.005). Administration of the vehicle of CBG (DMSO 0.1%) did not affect the firing rate of 5-HT cells (FR before DMSO: 0.95 ± 0.13 Hz vs*.* FR after DMSO: 1.01 ± 0.13 Hz, *n* = 9) (Figure 2C).

**FIGURE 3:**
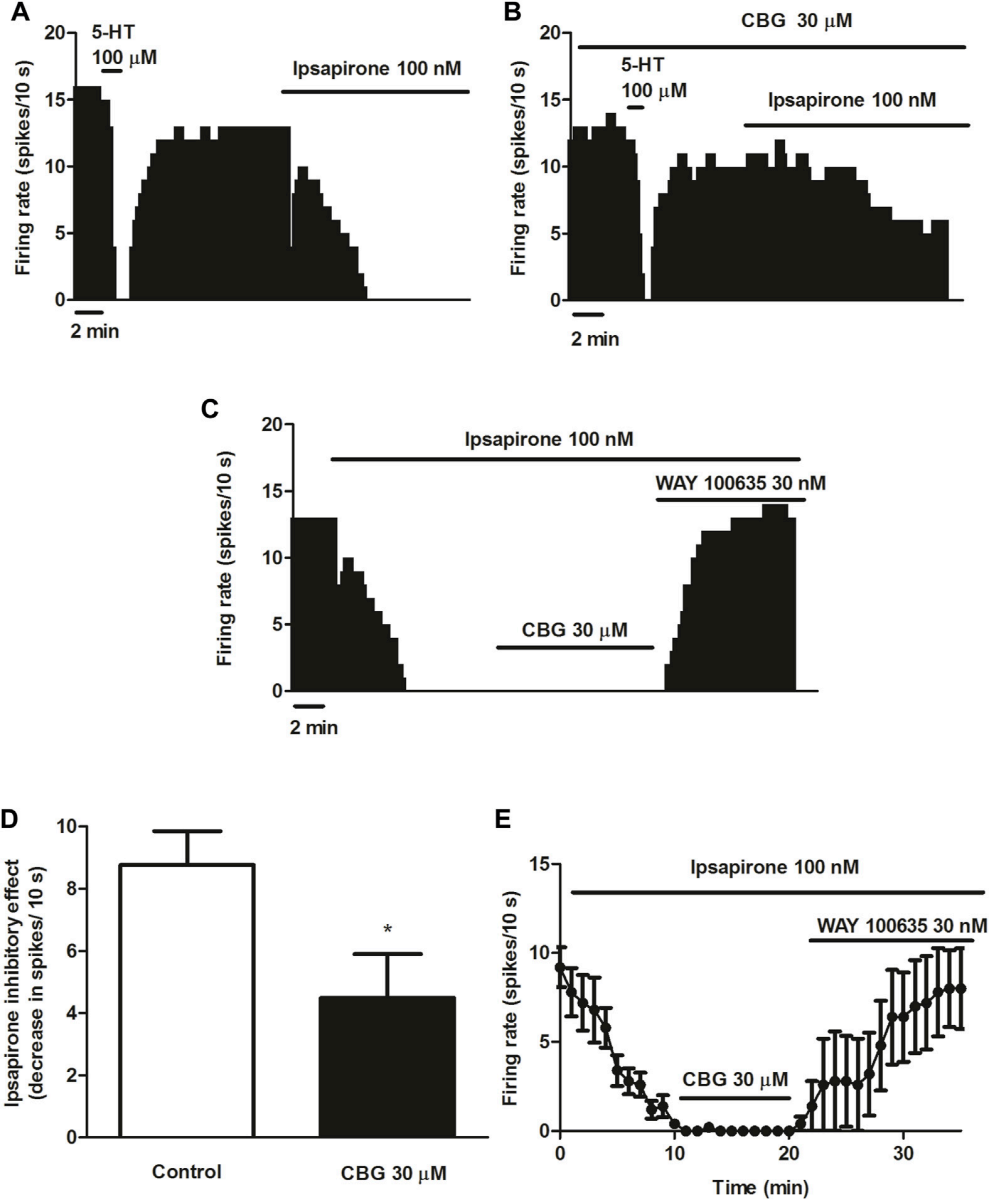
Effect of CBG on ipsapirone-induced inhibition of the firing activity of DRN 5-HT cells **(A–C)** Representative examples of firing rate recordings from DRN 5-HT cells, which show the inhibition of the firing activity of 5-HT neurons by ipsapirone (100 nM) **(A)**, the blockade of the inhibitory effect of ipsapirone (100 nM) by CBG **(B)** and the effect of CBG and WAY100635 on a neuron completely inhibited by ipsapirone **(C)**. The vertical lines refer to the integrated firing rate values (spikes per 10 s) and the horizontal lines represent the time scale. Drugs were perfused at the concentration and for the time indicated by the horizontal bars. **(D)** Bar histograms showing the inhibitory effect (decrease in spikes/10 s; mean ± SEM) induced by ipsapirone (100 nM) in the absence (*n* = 12) and the presence of CBG (30 μM, *n* = 9) **(E)** Time course of the firing rate of DRN 5-HT cells (mean ± SEM) in the presence of ipsapirone (*n* = 5), CBG (*n* = 5) or WAY100635 (*n* = 5). *p < 0.05 compared to the inhibitory effect induced by ipsapirone (100 nM) in the absence of CBG (30 μM) by an unpaired Student’s *t*-test.

5-HT activates different 5-HT receptors including the 5-HT_1A_, 5-HT_1B_ or 5-HT_7_ receptors. To avoid the action of 5-HT on non-5-HT_1A_ receptor, we used the more selective 5-HT_1A_ receptor agonist ipsapirone (100 nM), which has been studied due to its anxiolytic effects in several clinical trials. Administration of ipsapirone (100 nM, 10 min) completely inhibited the firing rate of DRN 5-HT cells (inhibitory effect: 98.4 ± 0.9%, *n* = 12) (Figures 3A, C–E). Thus, the firing rate after perfusion with ipsapirone was significantly lower than the firing rate before 5-HT_1A_ receptor agonist perfusion (FR before ipsapirone: 0.89 ± 0.11 Hz vs*.* FR after ipsapirone: 0.01 ± 0.01 Hz, *n* = 12, *p* < 0.005). In the presence of CBG (30 μM, 10 min), the inhibitory effect of ipsapirone (100 nM) on the firing rate of DRN 5-HT cells was reduced to 49.1 ± 13.8% (*n* = 9) (Figures 3B, D). The decrease of the firing rate produced by ipsapirone in the absence of CBG was significantly higher than that in the presence of the cannabinoid (decrease of the FR in the absence of CBG: 0.88 ± 0.11 Hz, *n* = 12 vs*.* decrease of the FR in the presence of CBG: 0.45 ± 0.14 Hz, *n* = 9, *p* < 0.05) (Figure 3D). It is known that a competitive 5-HT_1A_ receptor antagonist would restore the firing rate of a 5-HT neuron that has been previously inhibited by a 5-HT_1A_ receptor agonist. In order to study whether CBG mimicked the effect of a competitive 5-HT_1A_ receptor antagonist in the DRN, the cannabinoid was perfused for 10 min in 5-HT cells that were completely inhibited by ipsapirone. In these experiments, CBG (30 μM) did not reverse the inhibition induced by ipsapirone whereas perfusion with the 5-HT_1A_ receptor antagonist WAY100635 (30 nM) completely restored the firing rate of 5-HT cells to the initial firing rate value (Basal FR: 0.91 ± 0.11 Hz vs*.* FR after WAY100635: 0.98 ± 0.22 Hz, *n* = 5) (Figures 3C, E).

### 3.3 Effect of CBG on anxiety-like behavior in rats and the involvement of the 5-HT_1A_ receptor

The non-psychoactive CBD has been reported to produce anxiolytic effects through 5-HT_1A_ receptor-mediated mechanism in rodents (Resstel et al., 2009; Marinho et al., 2015; De Gregorio et al., 2019) and it has been suggested that modulation 5-HT_1A_ receptor of the DRN could be involved in its pharmacological effects (Rock et al., 2012; De Gregorio et al., 2019). The present study reveals that CBG shows similar pharmacological profile to that previously described for CBD at DRN 5-HT_1A_ receptor in rat brain slices (Mendiguren et al., 2022). Therefore, we studied whether CBG produced anxiolytic-like effects by different behavioral tests and then tested the putative involvement of the 5-HT_1A_ receptor.

**FIGURE 4:**
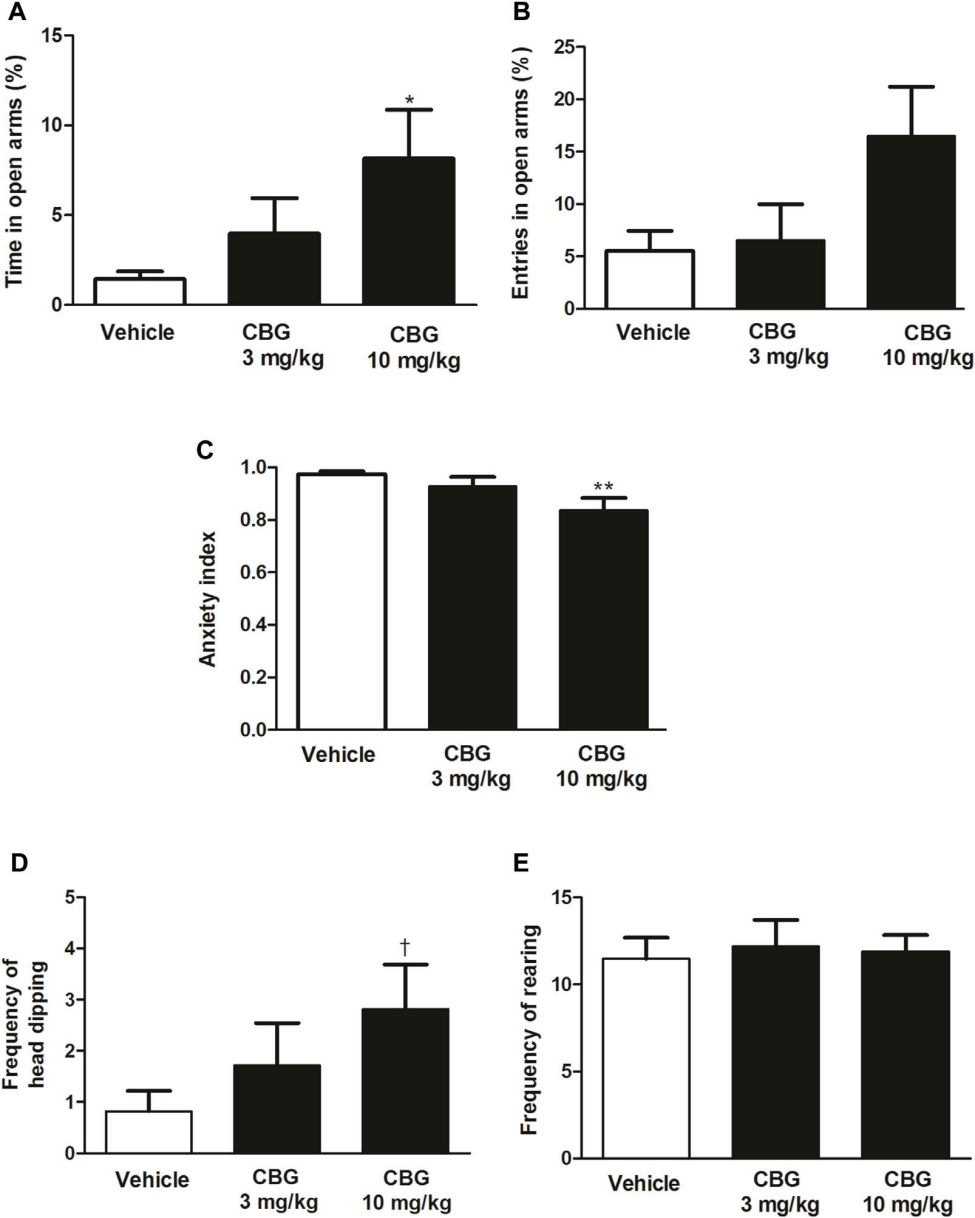
Effect of CBG on the anxiety-like behavior in rats by the EPMT **(A)** Percentage of time spent by the rats in the open arms, **(B)** percentage of open arms entries, **(C)** the anxiety index,**(D)** the frequency of head-dipping and **(E)** the frequency of rearing in rats injected with the vehicle (up to 10 mL/kg, i.p., *n* = 28), CBG (3 mg/kg, i.p., *n* = 21) and CBG (10 mg/kg, i.p., *n* = 11). Each bar represents the mean ± SEM of *n* animals. *p < 0.05 compared to percentage of time spent by the rats in the open arms in the vehicle-treated group and **p < 0.01 compared to the anxiety index value in the vehicle-treated group by the one-way ANOVA followed by Bonferroni’s Multiple Comparison Test. †*p* < 0.05 compared to the number of head-dipping in the vehicle-treated group by the Kruskal–Wallis test followed by Mann–Whitney *U* test.

First, the effect of acute administration of CBG (3 and 10 mg/kg, i.p.) was characterized in the EPMT. Our data revealed a significant increase in the percentage of time the rats spent on the open arms after administration of CBG (*p* < 0.05) and a trend towards an increase in the percentage of open arm entries (Figures 4A, B). Post hoc analysis showed that the significant effect was due to the dose of 10 mg/kg (vehicle group: 1.4 ± 0.5%, *n* = 28 vs*.* CBG 10 mg/kg group: 8.2 ± 2.7%, *n* = 11, *p* < 0.05). Furthermore, a significant lower anxiety index in the CBG-treated group was observed (*p* < 0.05). Post hoc analysis revealed that the reduction of anxiety index also occurs at the dose of 10 mg/kg (vehicle group: 0.97 ± 0.01, *n* = 28 vs*.* CBG 10 mg/kg group: 0.84 ± 0.05, *n* = 11, *p* < 0.01). These data suggest that acute injection of CBG (10 mg/kg, i.p.) produces an anxiolytic effect in rats. Injection of lower dose of CBG (3 mg/kg, i.p., *n* = 21) did not significantly change the percentage of time spent in the open arms, the open arms entries or the anxiety index compared to the vehicle-treated group (Figures 4A–C). No statistical differences were observed between CBG (3 mg/kg)-treated and CBG (10 mg/kg)-treated groups in the mentioned variables.

In addition, administration of CBG (10 mg/kg, i.p.), but not CBG (3 mg/kg, i.p.), significantly increased the frequency of head-dipping compared to the vehicle-treated group (vehicle group: 0.82 ± 0.39, *n* = 28 vs*.* CBG 10 mg/kg group: 2.82 ± 0.88, *n* = 11, *p* < 0.05), which is indicative of a reduction of anxiety-like behavior in cannabinoid-treated group (Figure 4D). CBG (3 and 10 mg/kg i.p.) did not change the frequency of rearing with respect to the control group (vehicle group: 11.89 ± 0.96, *n* = 28 vs*.* CBG 3 mg/kg group: 11.48 ± 1.23, *n* = 21; CBG 10 mg/kg group: 12.18 ± 1.54, *n* = 11) (Figure 4E).

**FIGURE 5:**
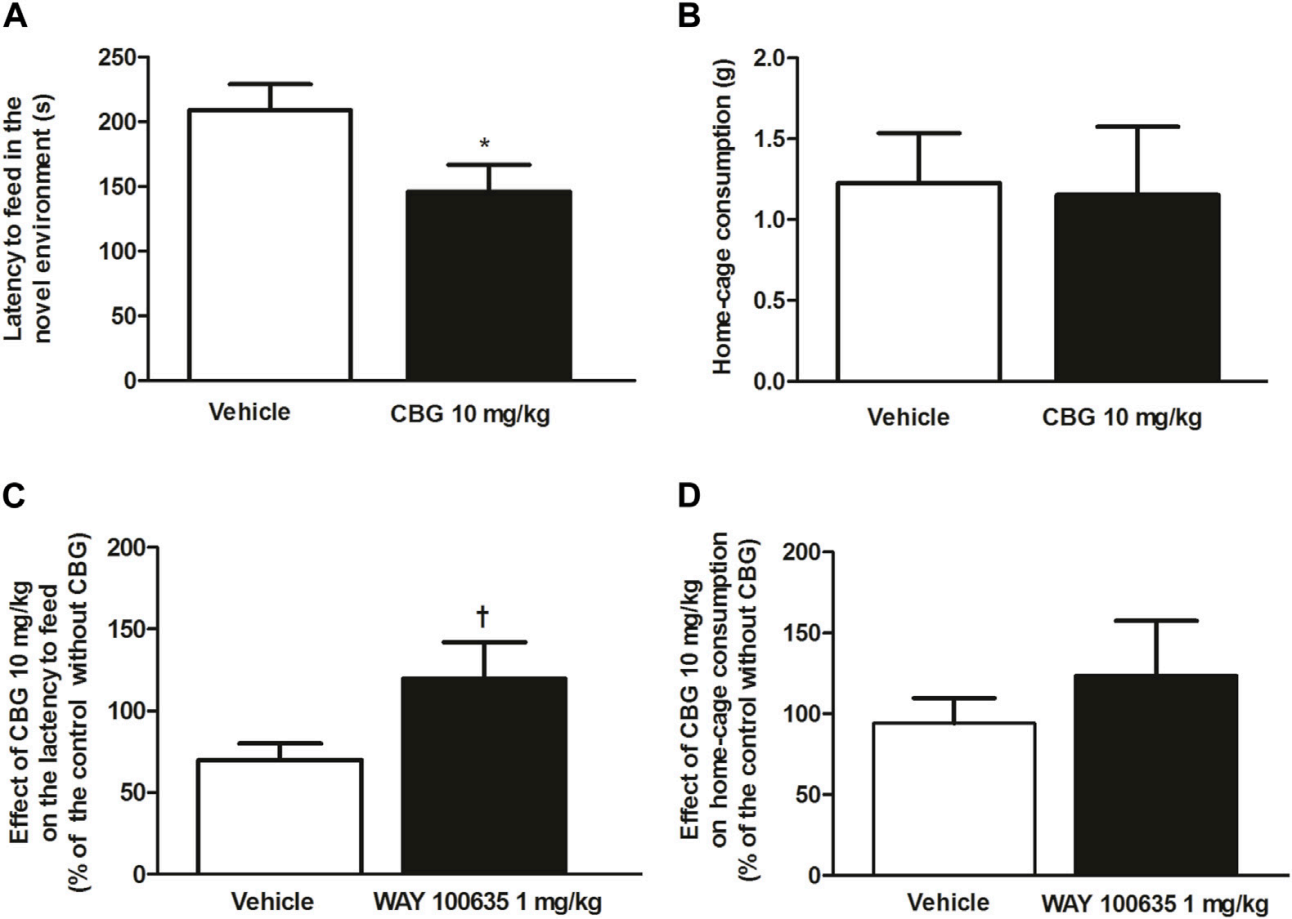
Effect of CBG on the anxiety-like behavior in rats by the NSFT and the involvement of 5-HT_1A_ receptor **(A)** Time latency to feed in the novel environment (s) and **(B)** home-cage consumption (g) in rats treated with the vehicle (up to 10 mL/kg, i.p., *n* = 20) or CBG (10 mg/kg, i.p., *n* = 13). Values are the mean ± SEM of *n* animals **(C)** Effect of CBG (10 mg/kg, i.p.) on the latency to feed and on home-cage consumption **(D)** in the absence of WAY100635 (1 mg/kg, i.p., *n* = 13) and in rats pretreated with WAY100635 (1 mg/kg, i.p., *n* = 12). Values are the mean ± SEM of the effect of CBG on the latency to feed or home-cage consumption in *n* animals, calculated as the percentage of the value of their corresponding control groups without CBG. *p < 0.05 compared to the time latency to feed in the vehicle-treated group by the unpaired Student’s *t*-test. †*p* < 0.01 compared to the effect of CBG in the absence of WAY100635 by the unpaired Student’s *t*-test.

After observing the anxiolytic-like effect produced by CBG (10 mg/kg, i.p.) on the EPMT, we performed the NSFT to characterize the action of the cannabinoid both in the novel environment and home-cage consumption. Acute administration of CBG (10 mg/kg, i.p.) significantly reduced the time until the first feeding event in the novel environment compared to the vehicle group (*n* = 13, *p* < 0.05) (Figure 5A), suggesting an anxiolytic-like effect. In fact, the rats treated with CBG took significantly less time to eat the food placed in the center of the field (vehicle group: 208.90 ± 20.32 s, *n* = 20; CBG group: 146 ± 20.83 s, *n* = 13; *p* < 0.05) (Figure 5A). Putative interaction of CBG with central targets could lead to stimulation of feeding behavior and yield confounding results in the NSFT. Therefore, to rule out differences in food intake between groups we measured the amount of food ingested by the animals when they were returned back to the home-cage. In the housing cage, consumption of food in CBG (10 mg/kg, i.p.)-treated group did not significantly differ from that of the vehicle-treated group (*n* = 13 and *n* = 20, respectively) (Figure 5B). Furthermore, no differences were observed in the time latency to eat in the CBG-treated group (98.15 ± 18.50 s) compared to that in the vehicle-treated group (87.94 ± 19.4 s). This suggests that the reduction of latency time until the first feeding event produced by CBG in the novel environment did not result from stimulation of feeding behavior.

In order to study if the effect of CBG was mediated by the 5-HT_1A_ receptor, the 5-HT_1A_ receptor antagonist WAY100635 (1 mg/kg, i.p.) was injected 30 min before administration of CBG (10 mg/kg, i.p.) and NSFT was performed. Treatment with WAY100635 (1 mg/kg, i.p.) by itself did not affect the time latency to feed (vehicle group: 208.90 ± 20.32 s, *n* = 20 vs*.* WAY100635 group: 225.80 ± 50.28 s, *n* = 13) or the home-cage consumption (vehicle group: 1.23 ± 0.15 g, *n* = 20 vs*.* WAY100635 group: 1.03 ± 0.23 g, *n* = 13). However, it increased the latency to eat in the home-cage (vehicle group: 87.94 ± 19.41 s vs*.* WAY100635 group: 225.10 ± 66.33 s). Pretreatment with WAY100635 (1 mg/kg, i.p.) significantly reduced the effect of CBG (10 mg/kg, i.p.) on the latency to feed in the novel environment Thus, in the absence of WAY100635, the effect of CBG (10 mg/kg, i.p.) on the latency to feed, which was estimated as the percentage of time latency to feed after CBG with respect to its corresponding control group (vehicle-treated group), was 69.91 ± 9.97% while in the presence of the 5-HT_1A_ receptor antagonist, the effect of CBG (10 mg/kg, i.p.) was 120.14 ± 21.90% of that in its control group (WAY100635 treated group) (*n* = 12, *p* < 0.05) (Figure 5C). No significant change in home-cage consumption was found after CBG (10 mg/kg, i.p.) injection in rats pretreated with WAY100635 (1 mg/kg, i.p.) (*n* = 12) with respect to that in the vehicle-treated group (*n* = 13) (Figure 5D). Similarly, the effect of CBG on the lactency to feed in the home-cage in rats pretreated with WAY100635 was not different from that in the group that was not injected with the 5-HT_1A_ receptor antagonist. Thus, in the absence of WAY100635, the effect of CBG on the latency to feed in the home-cage, which was estimated as the percentage of time latency to feed after CBG with respect to its corresponding control group (vehicle-treated group), was 111.60 ± 21.03%, whereas in the presence of WAY100635 the effect of CBG was 95.71 ± 30.43% of that in its control group (WAY100635-treated group). Our data suggest that the anxiolytic-like effect of CBG on NSFT was mediated by activation of 5-HT_1A_ receptor.

## 4 Discussion

The present work was carried out to characterize the effect of CBG on the neuronal activity of NA and 5-HT cells and on the anxiety-like behavior in rats. Our results show that CBG slightly reduces the basal firing rate of LC NA cells but fails to change the firing rate of DRN 5-HT cells. CBG reduces the inhibitory effect of the selective α_2_-adrenoceptor agonist UK14304 on LC NA cells and of the selective 5-HT_1A_ receptor agonist ipsapirone on DRN 5-HT cells, but fails to alter the inhibitory effect of the endogenous neurotransmitters NA and 5-HT. In addition, CBG does not reverse the inhibitory effect of ipsapirone on the firing rate of DRN 5-HT cells. Besides, our data reveal that CBG produces anxiolytic-like effects in rat by a 5-HT_1A_ receptor-mediated mechanism.

In this study, single-unit extracellular recording techniques in brain slices were used to study the regulation by CBG of 5-HT_1A_ and α_2_-adrenoreceptors-mediated effects on the firing rate of LC NA and DRN 5-HT cells in rats. *In vitro* techniques of cell recording allow to isolate somatodendritic responses of neurons to bath application of drugs. The concentrations and time of application of cannabinoids and 5-HT_1A_ and α_2_-adrenoreceptor agonists or antagonist were selected based on the reported Ki values for the receptors and their use in previous electrophysiological assays performed in monoaminergic nuclei from rat brain slices (Haj-Dahmane et al., 1991; Nörenberg et al., 1997; Corradetti et al., 1998; Mendiguren and Pineda, 2004; Grandoso et al., 2005; Jones et al., 2010; Mendiguren et al., 2022). For behavioral assays, the EPMT and NSFT were used to elucidate the effect of CBG on both the anxiolytic-like behavior and/or food consumption. CBG was administrated at the dose previously reported to produce an anxiolytic-like effect by the open-field test (Zagzoog et al., 2020) and at the time range in which the maximal concentration was achieved after i.p. injection in rodent (Deiana et al., 2012). Accordingly, the 5-HT_1A_ receptor antagonist WAY100635 was administrated at the dose and time point reported to block 5-HT_1A_ receptor-mediated anxiolytic effects in rodent (Bektas et al., 2020).

We observed that CBG induces a slight reduction of the firing rate of LC NA cells. A previous binding assay performed in mouse whole brain membranes has revealed that CBG behaves as a highly potent α_2_-adrenoceptor agonist (EC_50_ = 0.2 nM) (Cascio et al., 2010). In fact, stimulation of ^[35S]^GTPɣS binding induced by CBG was blocked by the α_2_-adrenoceptor antagonist yohimbine. Additionally, electrically-evoked contractions of mouse vas deferens have been observed to be inhibited by CBG, further pointing to agonistic properties of the cannabinoid at the α_2_-adrenoceptor (Cascio et al., 2010).

However, in previous electrophysiological assays performed in slices from the LC, α_2_-adrenoceptor agonists have been shown to elicit complete inhibition of the neuronal activity of NA cells at rather low concentrations (nM) (Chiu et al., 1995), which would have been expected to occur after CBG perfusion. Moreover, binding assays showing potent agonist action of CBG at the α_2_-adrenoceptor were performed in brainstem membranes preparation, which contain several brain regions with different type of cells that may account for the overall effect of the cannabinoid. Therefore, although an α_2_-adrenoceptor antagonist was not tested in our study, one could speculate that the slight reduction of the firing rate of LC cells produced by CBG (30 µM) could rather result from a non-α_2_-adrenoceptor-mediated mechanism.

Interestingly, we observed that CBG regulates α_2_-adrenoceptor-mediated inhibitory responses on the neuronal activity of LC cells since the effect of the selective α_2_-adrenoceptor agonist UK14304 was reduced, although not that of the endogenous adrenergic neurotransmitter NA. This fact could be due to the lack of selectivity of NA onto α-adrenoreceptors since activation of α_1_-adrenoreceptors could have masked α_2_-adrenoreceptor-mediated inhibitory responses. Additionally, the endogenous adrenergic neurotransmitter NA could have been rapidly reuptake from the synaptic cleft while the structurally different UK14304 could remain longer in the slice preparation. One possible hypothesis that would explain the regulation of selective α_2_-adrenoceptor agonist-induced inhibition of the firing rate by CBG would be an indirect (i.e., allosteric) interaction with the α_2_-adrenoceptor.

In our study CBG fails to affect the firing rate of DRN 5-HT cells but reduces the inhibitory effect of the 5-HT_1A_ receptor agonist ipsapirone in the DRN suggesting an antagonist action at the serotonergic receptor. Two approaches were used to test the effect of CBG on 5-HT_1A_ receptor-mediated responses in the DRN cells. On the one hand, we perfused ipsapirone, which has been shown to cause a complete inhibition of the firing rate of DRN neurons at the concentration used (Haj-Dahmane et al., 1991; Mendiguren et al., 2022), both in the absence and the presence of CBG. As previously stated, we observed that CBG partially prevented the inhibitory effect of ipsapirone. On the other hand, the same concentration of CBG was used to investigate if it could mimick the effect of a classical competitive antagonist of the 5-HT_1A_ receptor (i.e., WAY100635). Indeed, a potent competitive antagonist at 5-HT_1A_ receptor in the DRN (Corradetti et al., 1998) would reverse the inhibition produced by a selective 5-HT_1A_ receptor agonist in electrophysiological studies. Although few data exist on the interaction of CBG with the 5-HT_1A_ receptor, our results are partially in line with the reported blockade of 5-HT_1A_ receptor-mediated responses described by other authors *in vitro* (Cascio et al., 2010; Nachnani et al., 2021). Thus, CBG antagonized the effect of 5-HT_1A_ receptor agonist 8-OH-DPAT on ^[35S]^GTPɣS binding to mouse brain membranes (Cascio et al., 2010). However, Cascio et al. reported that CBG behaved as a neutral antagonist at 5-HT_1A_ receptor while our study rather points to a non-competitive antagonist at the 5-HT_1A_ receptor since CBG fails to reverse the inhibitory effect of ipsapirone on the firing activity of DRN 5-HT cells but the previously characterized competitive antagonist WAY100635 completely restores it. In fact, according to our results the effect of CBG on ipsapirone-induced inhibition of the firing rate of DRN 5-HT cells would not derive from its action onto the orthosteric site of the 5-HT_1A_ receptor but would rather result from a negative allosteric modulation of the receptor. An indirect modulation of the 5-HT_1A_ receptor has also been previously described for CBD (Rock et al., 2012; Galaj and Xi, 2021), one of the best studied non-psychoactive cannabinoid that shares pharmacological features with CBG (Nachnani et al., 2021). Thus, in brain slices from the DRN, CBD reduced the effect of 5-HT_1A_ receptor agonist by an indirect mechanism (Mendiguren et al., 2022). As previously mentioned, the reasons behind the discrepancy between binding data (competitive antagonist) and electrophysiological assays (non-competitive antagonist) could rely on the differences in the preparations used (brain membranes vs*.* slices), techniques (binding vs*.* electrophysiology) or species (rat vs*.* mouse).

Our data show that CBG fails to modify the inhibition induced by 5-HT on the DRN. This is in line with previously reported lack of effect of the non-psychoactive CBD on 5-HT-induced inhibition (Mendiguren et al., 2022), which could result from the ability of 5-HT to activate non-5-HT_1A_ receptors. Thus, ipsapirone has been shown to be selective for 5-HT_1A_ receptor where as 5-HT has been described to activate 5-HT_7_ receptors (Roberts et al., 2004; Albert and Vahid-Ansari, 2019) and the presynaptic 5-HT_1B_ receptors (Morikawa et al., 2000; Adell et al., 2001). 5-HT_7_ receptors modulate GABAergic neurons while some presynaptic 5-HT_1B_ receptors have been shown to behave as heteroreceptors to inhibit glutamatergic neurotransmission in the DRN (Lemos et al., 2006; Geddes et al., 2016). In addition, several evidence shows that 5-HT and selective 5-HT_1A_ receptor agonists may activate differently the 5-HT_1A_ receptor. On the one hand, there are neurons that are completely inhibited by ipsapirone but are not equally responsive to 5-HT. On the other hand, 5-HT and selective 5-HT_1A_ receptor agonist could also differ in coupling to adenylate cyclase (Varrault and Bockaert, 1992; Polter and Li, 2010).

In our study, CBG (10 mg/kg, i.p.) increases the time spent by the rats in the open arms or the number of head-dipping and reduces the anxiety index in the EPMT. In the NSFT, CBG decreases the time latency to feed in the novel environment. These are all indicative of an anxiolytic-like effect of the cannabinoid. Few studies have tested the anxiolytic effects of CBG in rodents and the reported results have been controversial. Thus, in agreement with our results, Zagzoog et al. demonstrated that acute administration of CBG produced anxiolytic-like effects in the open-field test in mice at the same dose, via and time point herein used (Zagzoog et al., 2020). In contrast, a very recent study has reported that injection of a single dose of CBG failed to produce anxiolytic effects in the light-dark test in mice (Zhou et al., 2022). However, in the latter study a mouse model of post-traumatic stress disorder was used, which could have accounted for a higher basal anxiety level of the animals and subsequent lack of effect of the cannabinoid.

With regard to previous data published in rats, it was reported that acute low doses of CBG (2.5 mg/kg, i.p.) failed to produce anxiolytic-like effects in the light-dark immersion test (O’Brien et al., 2013), which is in line with the lack of effect observed in our study at similar doses of CBG (3 mg/kg). In contrast to our results, other authors have reported that CBG did not produce anxiolytic effects even when given at higher doses in the open field test (Brierley et al., 2016). The discrepancy between the latter and our data could arise from the differences in the strain of rats (Lister Hooded vs*.* Sprague-Dawley) or route of administration (p.o vs*.* i.p.) used. In fact, Lister Hooded rats are known to be less responsive to anxiolytic drugs (McDermott and Kelly, 2008). Moreover, the amount of CBG reaching the site of action could have been substantially reduced after oral administration due to degradation.

However, at this point we cannot completely exclude other effects of CBG that could have contributed to the reduction of latency time to feed in the NSFT or to the increase in the time spent in the open arms in the EPMT observed in our study, such as alteration of motor activity. In view of previous authors data this seems unlikely to occur because no changes of locomotor activity have been reported at similar doses, via or administration time herein used in rodents (Brierley et al., 2016; Zagzoog et al., 2020). Furthermore, in our experiments CBG does not change the number of rearings in the EPMT, which have been related to alteration of motor activity (Cruz et al., 1994). Interestingly, we observed that CBG fails to alter food intake at the same dose that reduces the latency to feed in the novel environment, which is also in line with the lack of effect on food intake previously reported (Farrimond et al., 2012). In fact, both alteration of motor performance and hyperphagic effects of cannabinoids have been shown to occur mainly by activation of CB_1_ receptor (Farrimond et al., 2012; Polissidis et al., 2013) and low affinity at the cannabinoid receptor has been reported for CBG (Nachnani et al., 2021). Therefore, our findings further support an anxiolytic-like effect of CBG at the tested dose. At this stage, the putative involvement of the CB_2_ receptor in the effects of CBG could not be ruled out since the cannabinoid has been shown to target CB_2_ receptor (Navarro et al., 2018). Thus, previous evidence has reported the expression of CB_2_ receptor in several brain areas regulating emotional states (Kibret et al., 2022) and has described its role in anxiety-related behavior (García-Gutiérrez and Manzanares, 2011). Our study reveals that CBG decreases the time of first feeding event in the NSFT by a 5-HT_1A_ receptor-mediated mechanism. Involvement of the 5-HT_1A_ receptor in the *in vivo* effects of CBG has not been reported yet although 5-HT_1A_ receptor-mediated neuroprotective effect of CBG has been recently observed *in vitro* (Echeverry et al., 2021). Thus, in previous studies showing anti-inflammatory (Carrillo-Salinas et al., 2014; Valdeolivas et al., 2015; Burgaz et al., 2021), analgesic or anxiolytic properties of CBG (Zagzoog et al., 2020) the effect of a 5-HT_1A_ receptor antagonist was not tested. Given the fact that CBG shows similar pharmacological profile to CBD onto DRN 5-HT_1A_ receptors *in vitro* and that CBD produces anxiolytic (Resstel et al., 2009) and other effects (analgesic, antiepileptic, antidepressant) via 5-HT_1A_ receptor *in vivo* (Zanelati et al., 2010; Linge et al., 2016; Sartim et al., 2016; De Gregorio et al., 2019; Peng et al., 2022) we cannot rule out that CBG may regulate DRN 5-HT_1A_ receptor to mediate some *in vivo* effect (i.e., anxiolytic). Additionally, in view of our results revealing modulation of α_2_-adrenoceptor-mediated inhibitory effects by CBG in the LC, the involvement of this receptor in the anxiolytic effects of the cannabinoid should not be discarded. Considering that anxiolytic effects have been attributed to some drugs that regulate the α_2_-adrenoceptor (Garakani et al., 2020) it would be of interest to investigate the role of the α_2_-adrenoceptor in the *in vivo* effects of CBG.

In conclusion, CBG slightly affects the firing rate of LC NA cells but not that of DRN 5-HT cells and hinders both the inhibitory effect produced by selective α_2_-adrenoceptor agonist on the NA-LC cells and by 5-HT_1A_ receptor agonist on 5-HT-DRN cells *in vitro*. One possible hypothesis is an allosteric modulation of LC α_2_-adrenoceptor and DRN 5-HT_1A_ receptor by CBG. Furthermore, the data suggest that CBG produces anxiolytic effect in rat through a 5-HT_1A_ receptor-mediated mechanism. In the future, it would be interesting to study the possible contribution of the modulation of 5-HT_1A_ receptors located in the DRN to the *in vivo* effects of the non-psychoactive cannabinoids.

To our knowledge this is the first study in which the effect of CBG on the neuronal activity of 5-HT and NA cells *in vitro* was tested and the involvement of 5-HT_1A_ receptor in an *in vivo* effect induced by CBG was reported. To characterize the effect of CBG on the neuronal activity of LC and DRN could be interesting in view of the role of these nuclei in the modulation of emotional state, arousal or control of pain, functions that could be altered by cannabinoids (Lowry et al., 2008; McDevitt and Neumaier, 2011; Campion et al., 2016). Our study provides new insights into the effects of CBG on monoaminergic systems in the rat. However, more studies are needed to elucidate the effects of CBG on NA and 5-HT systems both *in vitro* and *in vivo* and to delve into the mechanisms by which the cannabinoid regulate 5-HT_1A_ and α_2_-adrenoceptors.

## Data Availability

The raw data supporting the conclusion of this article will be made available by the authors, without undue reservation.

## Abbreviations

ACSF, artificial cerebrospinal fluid; CBD, cannabidiol; CBG, cannabigerol; CNS, central nervous system; DMSO, dimethylsulphoxide; DRN, dorsal raphe nucleus; EPMT, elevated plus maze test; GABA, γ-aminobutyric acid; GIRKs, G protein-coupled inwardly rectifying potassium channels; 5-HT, serotonin; ipsapirone, 2-[4-[4-(2-pyrimidinyl)-1-piperazinyl]butyl]-1,2-benzisothiazol-3(2H)-one-1,1-dioxide; LC, locus coeruleus; NA, noradrenaline; NSFT, novelty-suppressed feeding test; PE, Phenylephrine; UK14304, 5-Bromo-6-(2-imidazolin-2-ylamino)quinoxaline tartrate; WAY100635, N-[2-[4-(2-Methoxyphenyl)-1-piperazinyl]ethyl]-N-2-yridinylcyclohexanecarboxamide.
